# A full-thickness defect: is closure the only way?

**DOI:** 10.1055/a-2409-0175

**Published:** 2024-09-25

**Authors:** Libo Quan, Qingfen Zheng, Lixia Zhao, Bingrong Liu, Dan Liu

**Affiliations:** 1 191599 Department of Gastroenterology and Hepatology, The First Affiliated Hospital of Zhengzhou University, Zhengzhou, China; 2 105821 Department of Gastroenterology and Hepatology, Second Affiliated Hospital of Harbin Medical University, Harbin, China; 3 Department of Gastroenterology and Hepatology, The First Affiliated Hospital of Zhengzhou University, Zhengzhou, China

Closure is one of the standard steps during endoscopic full-thickness resection (EFTR) 1 2 . Though many closure strategies have been applied, including endoclips, kissing sutures, and over-the-scope clip systems, it is still time-consuming and skill-dependent 3 . Here, we present a large full-thickness defect following EFTR that healed successfully without closure due to coverage by the connective tissue.

A 19-year-old woman had an elevated giant lesion 54 mm in diameter at the antrum of the stomach ( Fig. 1 , Fig. 2 ). After acquiring informed consent, a standard EFTR was performed ( Video 1 ). Originally, a nylon ligature in combination with clips was chosen to close the defect. The scheduled defect closure was extremely difficult to accomplish because the arms of the available clips could not bring both edges of the defect into alignment for closure. Despite the full-thickness resection performed, we noticed that the base was completely covered by the connective tissue and the great omentum, which still maintained the integrity of the gastric body ( Fig. 3 ). Subsequently, the decision to keep the defect open with the help of nasogastric decompression was made after obtaining additional informed consent. Follow-up endoscopy on postoperative day 3 revealed the base of the defect was strong enough by touching with forceps, and therefore the nasogastric tube was withdrawn ( Fig. 4 ). The patient resumed a liquid diet on postoperative day 4 and was discharged without experiencing any adverse events. Endoscopy at the 2-month follow-up revealed the satisfactory closure of the wall defect ( Fig. 5 ).

**Fig. 1:**
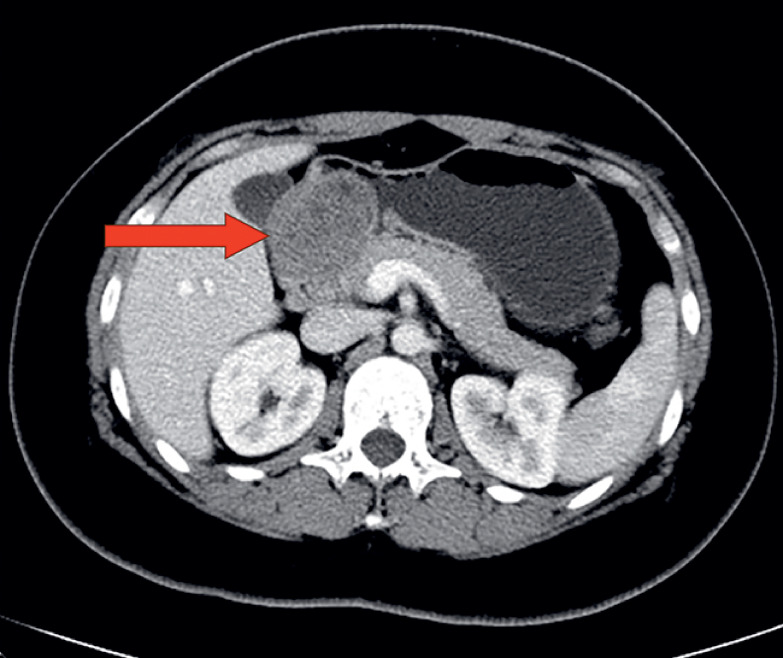
The submucosal tumor (red arrow) at the posterior wall of antrum on computed tomography.

**Fig. 2:**
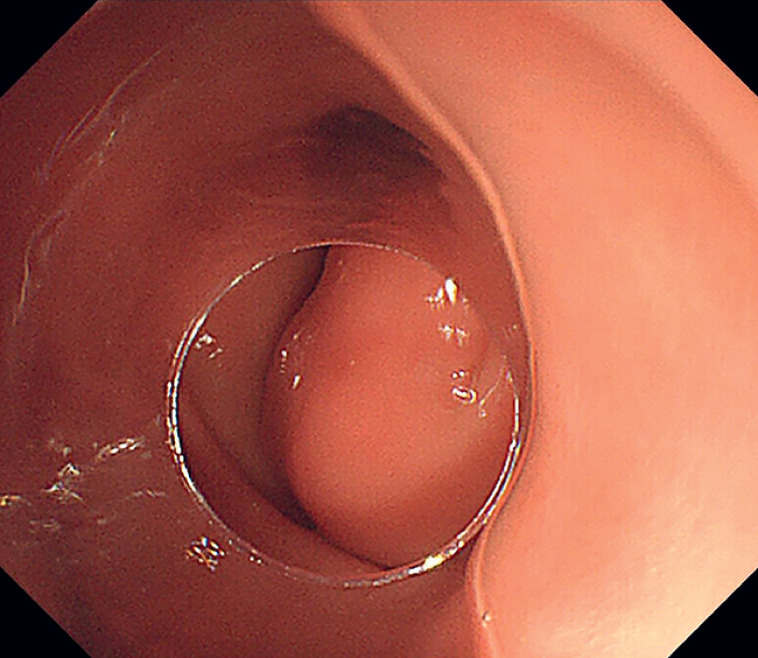
The tumor appearance with clear border on endoscopy.

**Fig. 3:**
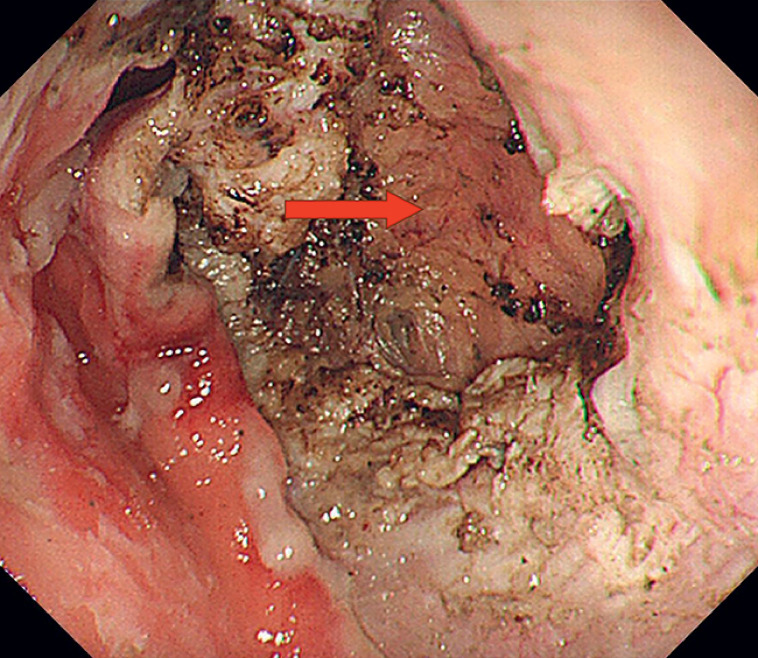
The defect was completely covered by connective tissue (red arrow) outside to keep the integrity of stomach.

**Fig. 4:**
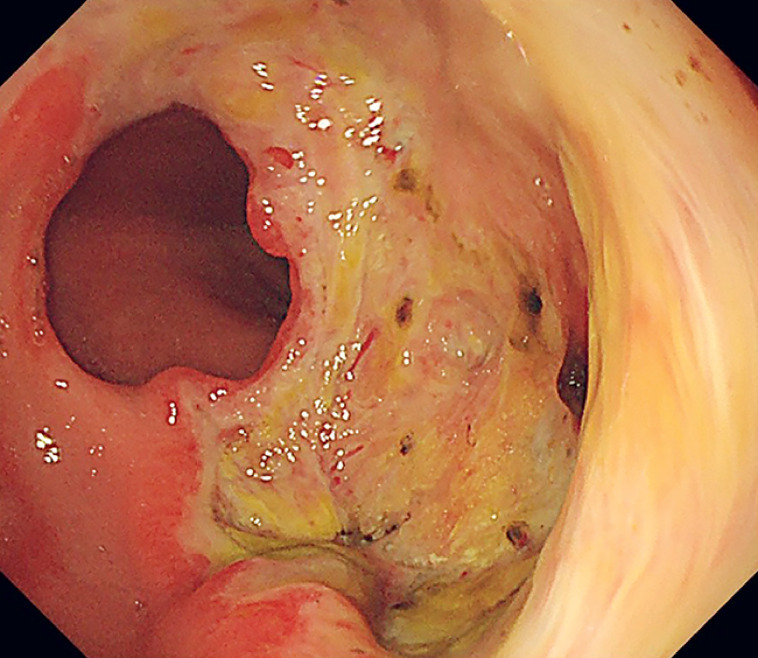
Endoscopic evaluation of the base on postoperative day 3.

**Fig. 5:**
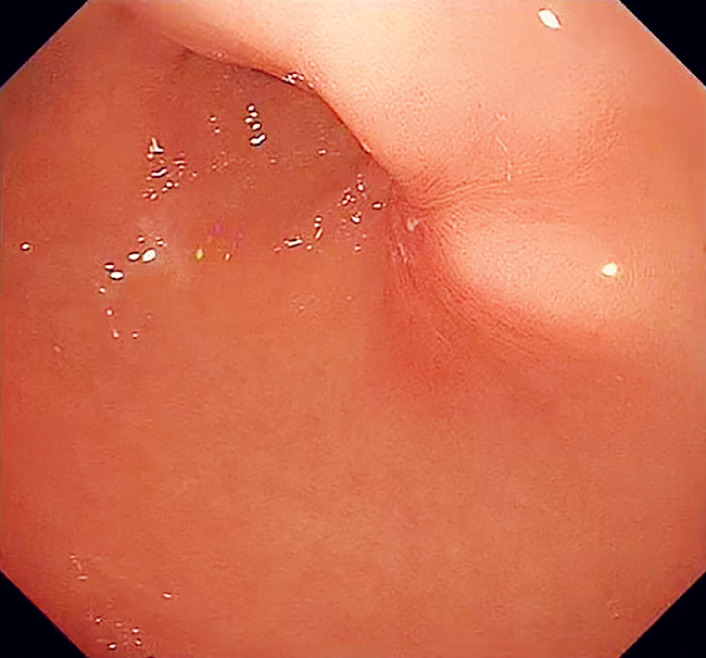
Endoscopy on 2-month follow-up revealed the satisfactory closure.

A giant submucosal tumor was resected by endoscopic full-thickness resection, and the non-leaking defect was not closed.Video 1

Our experience indicates that non-closure is sometimes an applicable choice under nasogastric decompression and close postoperative observation only if the defect is non-leaking. However, future data are definitely required to evaluate the safety and efficacy of non-closure.

Endoscopy_UCTN_Code_TTT_1AO_2AI

## Notes

Endoscopy E-Videos https://eref.thieme.de/e-videos
*E-Videos* is an open access online section of the journal *Endoscopy* , reporting on interesting cases and new techniques in gastroenterological endoscopy. All papers include a high-quality video and are published with a Creative Commons CC-BY license. Endoscopy E-Videos qualify for HINARI discounts and waivers and eligibility is automatically checked during the submission process. We grant 100% waivers to articles whose corresponding authors are based in Group A countries and 50% waivers to those who are based in Group B countries as classified by Research4Life (see: https://www.research4life.org/access/eligibility/ ). This section has its own submission website at https://mc.manuscriptcentral.com/e-videos .
